# A candidate gene investigation of methylphenidate response in adult attention-deficit/hyperactivity disorder patients: results from a naturalistic study

**DOI:** 10.1007/s00702-016-1540-7

**Published:** 2016-04-18

**Authors:** Tor-Arne Hegvik, Kaya Kvarme Jacobsen, Mats Fredriksen, Tetyana Zayats, Jan Haavik

**Affiliations:** 1 Department of Biomedicine, University of Bergen, 5009 Bergen, Norway; 2 K.G. Jebsen Centre for Research on Neuropsychiatric Disorders, University of Bergen, 5009 Bergen, Norway; 3 Center for Medical Genetics and Molecular Medicine, Haukeland University Hospital, 5021 Bergen, Norway; 4 Division of Mental Health and Addiction, Vestfold Hospital Trust, 3101 Tønsberg, Norway; 5 University of Oslo, 0318 Oslo, Norway; 6 Division of Psychiatry, Haukeland University Hospital, 5021 Bergen, Norway

**Keywords:** ADHD, Pharmacogenetics, ADRA2A, Methylphenidate, Stimulants

## Abstract

Attention-deficit/hyperactivity disorder (ADHD) is a common childhood onset neuropsychiatric disorder with a complex and heterogeneous symptomatology. Persistence of ADHD symptoms into adulthood is common. Methylphenidate (MPH) is a widely prescribed stimulant compound that may be effective against ADHD symptoms in children and adults. However, MPH does not exert satisfactory effect in all patients. Several genetic variants have been proposed to predict either treatment response or adverse effects of stimulants. We conducted a literature search to identify previously reported variants associated with MPH response and additional variants that were biologically plausible candidates for MPH response. The response to MPH was assessed by the treating clinicians in 564 adult ADHD patients and 20 genetic variants were successfully genotyped. Logistic regression was used to test for association between these polymorphisms and treatment response. Nominal associations (*p* < 0.05) were meta-analysed with published data from previous comparable studies. In our analyses, rs1800544 in the *ADRA2A* gene was associated with MPH response at a nominal significance level (OR 0.560, 95 % CI 0.329–0.953, *p* = 0.033). However, this finding was not affirmed in the meta-analysis. No genetic variants revealed significant associations after correction for multiple testing (*p* < 0.00125). Our results suggest that none of the studied variants are strong predictors of MPH response in adult ADHD as judged by clinician ratings, potentially except for rs1800544. Consequently, pharmacogenetic testing in routine clinical care is not supported by our analyses. Further studies on the pharmacogenetics of adult ADHD are warranted.

**Electronic supplementary material:**

The online version of this article (doi:10.1007/s00702-016-1540-7) contains supplementary material, which is available to authorized users.

## Introduction

Attention-deficit/hyperactivity disorder (ADHD) is a neuropsychiatric disorder characterized by developmentally inappropriate levels of impulsivity, hyperactivity and inattention (American Psychiatric Association 2013). Most ADHD patients are diagnosed in childhood and the symptoms are thought to persist into adulthood in its full syndromic form in approximately 35 % of the patients. However, more than 70 % of the patients may be burdened by ADHD symptoms or experience ADHD-related functional impairment as adults (Biederman et al. 2010, 2012).

The first-line pharmacological option in the treatment of ADHD in children and adults is methylphenidate (MPH) (National Institute for Health and Clinical Excellence 2008; Norwegian Directorate of Health 2014). MPH has been shown superior to placebo in providing symptom relief in ADHD (Epstein et al. 2014; Faraone and Buitelaar 2010), and is believed to exert its effect by inhibiting the dopamine transporter (encoded by *DAT/SLC6A3* gene) and/or the noradrenaline transporter (encoded by *NET/SLC6A2* gene) (Engert and Pruessner 2008). Although the exact mechanisms of MPH’s effects remain unknown, the inhibition of these transporters is thought to affect the extracellular concentrations of dopamine and noradrenaline in the brain, predominantly in the prefrontal cortex and striatum, and thus, moderate the symptoms of ADHD (Arnsten and Pliszka 2011; Engert and Pruessner 2008).

The response rates to MPH among adult ADHD patients range from 25 to 78 % in controlled trials (Wilens et al. 2011). A portion of this variability is likely explained by the definition of response, diagnostic criteria, sample characteristics, drop-outs and dosing parameters (Fredriksen et al. 2013; Wilens et al. 2011). In addition, some patients experience adverse effects that outweigh the therapeutic impact, although serious adverse effects are considered rare (Fredriksen et al. 2013). Genetic variations may contribute to variability in MPH response. Previous studies have investigated several single nucleotide polymorphisms (SNPs) and variable tandem repeats (VNTRs), mostly in or near genes involved in the dopamine and noradrenaline systems, as well as in genes encoding MPH metabolizing enzymes (Bruxel et al. 2014). Still, most reports have been negative for any association, or the results have been conflicting (Bruxel et al. 2014; Kambeitz et al. 2014).

One explanation for the lack of consistent pharmacogenetic findings could be the polygenic and multifactorial nature of ADHD, with genetic variants contributing small effects to its aetiology and, consequently, requiring large sample sizes in order to detect such effects. An additional potentially important factor may be the phenotypic heterogeneity of ADHD. Moreover, the symptoms of ADHD tend to change with age with most studies reporting a more pronounced decline in the hyperactivity and impulsivity symptoms, as compared to the inattention symptoms (Biederman et al. 2010, 2012; Pingault et al. 2015). MPH may not be effective against all ADHD symptoms to the same extent and the effect of MPH may change over time. Thus, pharmacogenetic studies conducted in ADHD children may not be directly applicable to adults. Despite this lack of reliable genetic markers in the published literature and the uncertainties regarding the impact of aging, several commercial genetic tests are being marketed to patients, parents and health care providers to guide pharmacological intervention in ADHD. Examples of such commercial tests include “Harmonyx^®^ Test for ADHD” (Harmonyx Diagnostics) and “GeneSight^®^ ADHD” (Assurex Health).

Discovering and affirming genetic variants involved in the response to MPH in ADHD patients could help guide treatment strategies with respect to symptom control and adverse effects, thus helping to secure treatment compliance and adherence (Gajria et al. 2014). This may be of great importance for adult ADHD patients, who often are burdened with polypharmacy, comorbidity and socioeconomic distress (Halmoy et al. 2009). In addition, identification of pharmacogenetic variants could provide greater insight into the pharmacodynamics of MPH and the biology of ADHD. In this study, we therefore, explored MPH response in an adult ADHD sample in the light of genetic variants that have previously been associated with changes in MPH response. We also hypothesized that variants in the genes formerly linked to psychiatric or pharmacological phenotypes and which are involved in MPH metabolism or MPH pharmacodynamics, could potentially affect the response to MPH in adult ADHD patients.

## Patients and methods

### Patient recruitment and assessment of MPH response

Participants were recruited as part of a large study on adult ADHD risk factors (Halmoy et al. 2009; Johansson et al. 2008). DNA was extracted from blood or saliva samples collected from clinically diagnosed adult ADHD patients of Norwegian descent all over Norway. The diagnosis of ADHD in adults was based on the Diagnostic and Statistical Manual of Mental Disorders IV (DSM-IV) criteria. The recruitment protocol has previously been described in detail elsewhere (Halmoy et al. 2009; Johansson et al. 2008).

The patients’ response to MPH was assessed by two questionnaires that were filled out by the patients’ treating physicians, mainly psychiatrists (but also some general practitioners). The questions were designed to reflect treatment decisions in routine clinical settings.

In Questionnaire 1, the physicians rated the effect of “Ritalin” and/or “Concerta” which represented immediate release MPH and extended release MPH, respectively (at the time Questionnaire 1 was made and first utilized, “Ritalin” was the only immediate release formulation and “Concerta” the only extended release formulation of MPH available in Norway). The treatment response was rated with the options: “Very good”, “Good”, “Has had effect, but discontinued due to side effects” and “Not sure or none”. The first three categories were grouped as MPH responders and “Not sure or none” were defined as MPH non-responders. Patients who had tried both “Ritalin” and “Concerta”, had to be rated as “Not sure or none” on both items to be defined as MPH non-responders. A minority of the patients (13 %) were scored on a slightly modified version of the questionnaire, Questionnaire 2, where the effect of MPH was rated directly and the response option “Has had effect, but discontinued due to side effects” was omitted. Here, the first two alternatives were used to define MPH responders and the third non-responders.

The physicians filling out the questionnaires also stated the patients’ date of birth and the date the questionnaires were signed. These data were used to calculate the age of the patients at the time of questionnaire submission.

The original questionnaire (Questionnaire 1) was developed, and later revised (Questionnaire 2), by the last author of this study, Jan Haavik, together with co-workers during the planning of the larger study on ADHD risk factors (Halmoy et al. 2009; Johansson et al. 2008).

### Selection of genetic variants

To identify genetic polymorphisms (SNPs and VNTRs) previously associated, either experimentally or theoretically, with MPH response in child or adult ADHD patients, we conducted a literature search in PubMed, The Pharmacogenomics Knowledge Base and Google Scholar. In addition, variants in genes involved in ADHD pathophysiology or MPH metabolism and shown to have pharmacogenetic effects were included.

### Genotyping and its quality control

All SNPs were genotyped at CIGENE facility at the Norwegian University of Life Sciences (Ås, Norway), with a Sequenom MassARRAY iPLEX system (Sequenom, San Diego, CA, USA) as described previously (Johansson et al. 2010). Variants failing genotyping with the Sequenom MassARRAY iPLEX system, were genotyped as part of a larger genotyping effort using Illumina HumanExome BeadChip 12v1_B (Zayats et al. manuscript in preparation). VNTRs were genotyped by polymerase chain reaction followed by fragment analysis as previously described (Johansson et al. 2008).

The VNTR alleles were dichotomized based on the literature: a specific number of repeats was defined as the risk allele and all others as non-risk. For *DRD5*, the148 bp-repeat allele was defined as the risk allele (Tahir et al. 2000), the 10-repeat allele (10R) was defined as the risk allele for *SLC6A3/DAT1* (Kambeitz et al. 2014) and the 7-repeat allele (7R) as the risk allele for *DRD4* (Hamarman et al. 2004).

In the analyses presented, genotyped SNPs passed quality control criteria of minor allele frequency (MAF) over 1 %, missingness below 10 % and Hardy–Weinberg equilibrium test *p* value above 0.005. SNP genotype missingness per individual was set to 15 %.

All included VNTRs demonstrated MAF over 1 %, missingness below 1 % and passed Hardy–Weinberg equilibrium test *p* value above 0.005 after dichotomization. VNTR genotype missingness per individual was set to a maximum of one out of three variants.

### Genetic association between selected genetic variants and MPH response

Genetic association between chosen genetic variants and MPH response was tested in the form of binary logistic regression implemented in R-software. Sex and age were included as covariates. Both dominant and additive models were examined. Multiple testing was controlled by Bonferroni method.

### Meta-analysis with previous studies

Genetic variants exhibiting signs of association (*p* < 0.05) were further explored in meta-analysis with the previously published studies. For this step, published data was screened with regard to comparability, meaning studies on adult ADHD patients (17 years of age or more) of European ethnicity with MPH response assessed with a binary outcome and the variants of interest having a similar allele distribution to our material.

Once publications suitable for meta-analysis were identified, reported genetic data were re-examined for association with MPH response together with our data by means of Fisher’s exact test. Meta-analysis was carried out using inverse-variance method in a random effects model directly from the counts data. Between-study heterogeneity was assessed by *I*^2^ measure. Analyses were performed in R-software, using Metafor 1.9-4 package (Viechtbauer 2010). Multiple testing was controlled by Bonferroni method.

## Results

### Selection of genetic variants, genotyping and its quality control

Overall, 20 genetic variants were selected: 17 SNPs and 3 VNTRs in 11 genes (Supplementary Table 1 describes the polymorphisms). All were successfully genotyped.

After quality control, there were 564 unique patients, of whom 487 were classified as MPH responders and 77 as MPH non-responders (Table 1). The number of patients for the different genotyping platforms is presented in Supplementary Table 2.

**Table 1:** Description of the examined sample of adult ADHD patients

	Questionnaire 1	Questionnaire 2	Total
Demographics			
Sex: male/female	247/246	24/47	271/293
Mean age (SD)	34.0 (10.1)	34.9 (9.7)	34.11 (10.0)
MPH effect			
Responder (%)	427 (86.6)	60 (84.5)	487 (86.3)
Non-responder (%)	66 (13.4)	11 (15.5)	77 (13.7)
Total	493	71	564

*MPH* methylphenidate, *SD* standard deviation

Questionnaire 1: Patients with MPH response rated as “Very good”, “Good” or “Has had effect, but discontinued due to side effects” defined Responders. “Not sure or none” defined Non-responder

Questionnaire 2: Patients with MPH response rated as “Very good” or “Good” defined Responders. “Not sure or none” defined Non-responders

### Genetic association between selected genetic variants and MPH response

All results of the association analyses are presented in Supplementary Table 3. In summary, prior to correction for multiple testing, one nominally significant (*p* < 0.05) finding in the alpha-2-adrenergic receptor 2A gene (*ADRA2A*) was noted. Namely, the G allele of rs1800544 (MspI −1291C>G), located in the 5-untranslated region (5′UTR), was associated with MPH non-response in a dominant model (OR 0.560, 95 % CI 0.329–0.953, *p* = 0.033).

No genetic variations were associated with MPH response after correction for multiple testing (*p* < 0.00125).

### Meta-analysis with previous studies

In total, one variant in a dominant genetic model, rs1800544, was associated with MPH response prior to Bonferroni correction for multiple testing and included in the meta-analysis. After screening the previously identified literature with our criteria, only one study was performed on adult ADHD patients of European descent with dichotomous assessment of MPH response (Contini et al. 2011) and, thus, suitable for meta-analysis together with our data. The threshold of significance was set to *p* < 0.00122.

The meta-analysis showed no significant effect of rs1800544 on MPH response (OR 0.711, 95 % CI 0.410–1.232, *p* = 0.251) (Table 2).

**Table 2:** Results of meta-analysis of rs1800544 genotypes and MPH response

Study (observed G-allele frequency^a^)	rs1800544 genotype	MPH responders	MPH non-responders	OR (95 %CI)	Crude *p* value*	*I* ^2^
Contini et al. (0.33)	GG+GC	67	13	1.069 (0.398–2.824)	1.000	
	CC	53	11			
This study (0.27)	GG+GC	194	39	0.587 (0.333–1.024)	0.048	
	CC	229	27			
Meta-analysis				0.711 (0.410–1.232)	0.251	24.17 %

OR >1 means that the variant is associated with MPH response and OR <1 that the variant is associated with MPH non-response

*MPH* methylphenidate, *OR* odds ratio, *95 %* *CI* 95 % confidence interval, *I* ^*2*^heterogeneity measure

* Threshold for significance after 40 + 1 tests *p* = 0.00122

^a^In both MPH responders and MPH non-responders

## Discussion

Knowledge about the pharmacogenetics of MPH response may increase our insights into the aetiology of ADHD and improve patient care. We therefore assessed the genetic background of MPH response in patients with adult ADHD.

One SNP, rs1800544, in the 5′UTR of the *ADRA2A* gene revealed nominal association with MPH response in adult ADHD patients. Located in a transcription factor binding site, rs1800544 may affect gene expression and, consequently, MPH’s pharmacological effect on the symptoms of ADHD (Xu and Taylor 2009). *ADRA2A* encodes an adrenergic receptor located pre- and post-synaptically in both the central and peripheral nervous system. It has been found to be highly expressed in brain areas that have been associated with ADHD (Arnsten and Pliszka 2011). In addition, electrophysiological studies have shown that MPH may affect cortical excitability through ADRA2A, which might partly explain MPHs effect on the symptoms of ADHD (Andrews and Lavin 2006). The selective ADRA2A agonist guanfacine is used in the treatment of ADHD, consistent with a potential involvement of *ADRA2A* in the pathophysiology of the disorder (Arnsten and Pliszka 2011). It is noteworthy, however, that meta-analysis of this variant with a comparable Brazilian study (Contini et al. 2011), revealed no association. This observation may be explained by a number of reasons: (a) the sample size of the Brazilian study is only approximately one-third of ours, (b) possible genetic admixture as the reported MAF in the Brazilian study is higher than that reported for the CEU population (Northern and Western Europeans from Utah, UT, USA) and more comparable to that of the MXL population (Mexican Ancestry in Los Angeles, CA, USA) (Abecasis et al. 2012) and (c), the phenotypic heterogeneity of ADHD. Thus, meta-analysis with a larger and more homogeneous sample is warranted.

Previously, the G allele of rs1800544 has been noted to be associated with the improvement of MPH response in children and adolescents (Cheon et al. 2009; da Silva et al. 2008; Polanczyk et al. 2007). However, negative studies have also been reported in children and adolescents (Kim et al. 2011; Park et al. 2013) as well as in adults (Contini et al. 2011). Interestingly, in our study of adult ADHD, the G allele was associated with MPH non-response. As the symptomatology of ADHD is thought to change with age (Biederman et al. 2010, 2012; Faraone et al. 2006; Pingault et al. 2015), it is difficult to draw firm conclusions regarding the exact effect of this allele. There may be age-related changes in human neurobiology, such as alterations in gene expression patterns (Shingai et al. 2014).

In general, pharmacogenetic findings in paediatric ADHD samples reveal little to no replication in adults. This absence of replication may be explained by age-related changes in ADHD symptomatology, as well as likely variability in MPH effect itself. It has been reported in some studies that, compared to inattention, MPH has greater impact on the symptoms of hyperactivity and impulsivity in children (Beery et al. 2013). As inattention is believed to become more pronounced in the adult form of ADHD, pharmacological effects of MPH on attention may be more important in this group. Consequently, the genetic variants mediating the pharmacogenetic effects of MPH seen in children may not be of the same importance in adults. Nonetheless, randomised controlled studies in adults indicate that MPH is as effective on inattention symptoms as it is on hyperactivity and impulsivity (Epstein et al. 2014; Spencer et al. 2005).

Pharmacogenetic testing is being introduced for a number of neuropsychiatric disorders, including ADHD (Assurex Health; Dediemar 2015; Harmonyx Diagnostics; Hicks et al. 2015). Based on our results, such testing in adult ADHD for any of the variants assessed in our study is not justified, as only a single variant was nominally associated with MPH response. Additionally this effect was in the opposite direction of what has previously been described in studies conducted in children and adolescents.

This study should be viewed in the light of its limitations. Even with 564 participants, which to our knowledge is the largest pharmacogenetic study in ADHD, our study is still small and relatively underpowered to detect the small effect sizes of common variants.

Another limitation of our study may be phenotypic heterogeneity. Since psychiatric comorbidities, including substance abuse as well as polypharmacy, are common among our recruited adult ADHD patients (Halmoy et al. 2009), the MPH response observed in this study is likely to be affected by these factors (Retz and Retz-Junginger 2014). However, the presence of comorbidities may better approximate the average ADHD patient encountered in routine clinical practice (Sobanski et al. 2007). Furthermore, a recent naturalistic survey on instant release MPH concluded that psychiatric comorbidities may not be important predictors of medication response (Victor et al. 2014).

The overall treatment response was assessed by clinicians using two slightly different variants of a questionnaire. It is unlikely that there were systematic differences in how the physicians filled out the questionnaires, as the percentage of treatment responders was very similar in both (Table 1). Moreover, since this is an observational study, observer-expectancy effects might be present. The response rating schemes used in this study were designed to mirror routine clinical judgement, which is usually based on symptom scores and additional information provided by the patients. Thus, such clinical ratings are not directly comparable to the response scores derived from single symptom measurements or neuropsychological tests. In addition, laboratory recorded phenotypes and symptom rating scales may only capture minor phenotype changes and core symptom reduction (Beery et al. 2013). In contrast, our phenotype may better reflect changes in the patients’ everyday function.

A substantial proportion of our sample was recruited through a registry of adult ADHD patients who applied for stimulant treatment (Johansson et al. 2008). Therefore, some recruitment bias could be present in our sample. It may be that our patients had more severe ADHD symptoms as compared to the “average” ADHD patients, and severity has been shown to be a positive predictor of MPH response (Buitelaar et al. 2011; Victor et al. 2014). However, the patients with the greatest symptom burden are also the most functionally impaired, and would therefore perhaps be the ones that could benefit the most from accurate genetic guidance of pharmacological therapy.

Finally, the strength of the association signals presented in the previous pharmacogenetic studies also affect the outcomes of this study. For instance, as several of the previously reported variants have only been tested in small and underpowered studies, false positive findings are likely. Moreover, some of the variants investigated in this study were tested for association with clinical response to MPH for the first time.

In summary, none of the examined variants reached the threshold for statistical significance after correction for multiple testing. The SNP rs1800544 in *ADRA2A*, revealed nominally significant association, but the direction of effect was the opposite to that reported in previous studies conducted in children and adolescents. As some of the examined variants represent the existing pharmacogenetic markers of MPH, also used in commercial tests, our results suggest that such tests cannot reliably predict treatment response in adult ADHD patients. Therefore, more research is warranted before these markers can be recommended for routine clinical use.

## Electronic supplementary material

Below is the link to the electronic supplementary material.

Supplementary material 1 (pdf 217 kb)
