# Supplementary material for: A candidate gene investigation of methylphenidate response in adult attention-deficit/hyperactivity disorder patients: results from a naturalistic study
Source: J Neural Transm (Vienna). 2016 Apr 18;123:859–65. doi: 10.1007/s00702-016-1540-7 (PMC4969350; doi:10.1007/s00702-016-1540-7)
Supplement: Supplementary file 1 — Supplementary material 1 (pdf 217 kb) [file 702_2016_1540_MOESM1_ESM.pdf]

A candidate gene investigation of methylphenidate response in adult attention-deficit/hyperactivity disorder patients: results from a naturalistic study

Tor-Arne Hegvik, Kaya Kvarme Jacobsen, Mats Fredriksen, Tetyana Zayats, Jan Haavik

Corresponding author:

Tor-Arne Hegvik

Department of Biomedicine, University of Bergen, 5009 Bergen, Norway

K.G. Jebsen Centre for Research on Neuropsychiatric Disorders, University of Bergen, 5009 Bergen, Norway

E-mail address: tahegvik@gmail.com

Supplementary table 1. Description of the analysed genetic variants

Gene	SNP	Reference and reason for inclusion in this study
GRM7	rs3792452	One of the top SNPs in a GWAS on MPH effect in children where the G allele was associated with better a response to MPH (Mick et al. 2008). The SNP is located in a gene expressed in brain areas associated with ADHD and was therefore judged to be relevant by the authors of the study.
DRD5	18.5 kb 5-prime VNTR	148bp allele is over-transmitted in Turkish children who are MPH responders (Tahir et al. 2000) .
LPHN3	rs6551665	G allele has been associated with increased stimulant response in children (Arcos-Burgos et al. 2010), though in a later study in children it was associated with a worse MPH response (Labbe et al. 2012)
LPHN3	rs6858066	G allele has been associated with a good MPH response in children (Labbe et al. 2012)
LPHN3	rs2345039	G allele has been associated with a poor MPH response in children (Labbe et al. 2012)
SLC6A3/DAT1	rs2963238	A allele has been associated with a good response to the antipsychotic clozapine in schizophrenia patients (Xu et al. 2010).
SLC6A3/DAT1	rs2652511	C allele has been associated with both child and adult ADHD (de Azeredo et al. 2014).
SLC6A3/DAT1	3'UTR VNTR	A meta-analysis including both children and adults indicates that there are no, or possibly a minor effect from the 10-repeat allele (10R). The minor effect was noted in naturalistic studies where 10R homozygosity predicted a reduced effect from MPH on ADHD symptoms (Kambeitz et al. 2014).
ADRA2A	rs1800544<br>(MspI)	The G allele has been associated with increased improvement in inattentiveness in children and adolescents using MPH (da Silva et al. 2008) and symptomatically good response to MPH in children (Cheon et al. 2009). Though the results from both children and adults are conflicting (Contini et al. 2011; Kim et al. 2011; Park et al. 2013).
ADRA2A	rs553668<br>(DraI)	The variant has been shown to interact with the *DRD4* VNTR and affect the MPH response in children (Hong et al. 2012). However, the SNP alone has not been associated with changes in the MPH response in neither children or adults (Contini et al. 2011; Kim et al. 2011; Park et al. 2013)
DRD4	Exon 3 VNTR	4-repeat allele has been associated with more favourable MPH dose-response curve in children (Froehlich et al. 2011) and conversely the 7-repeat allele has been shown to predict higher doses of MPH necessary for satisfactory response in children (Hamarman et al. 2004). The 4-repeat allele has also been associated with a good response to MPH in Korean children (Cheon et al. 2007). A study in adults has not shown any effect (Contini et al. 2012).

BDNF	rs6265	The G allele has been associated with a better response to MPH in Korean children (Kim et al. 2011).
BDNF	rs61888800	The G allele has been associated with antidepressant response in adults (Licinio et al. 2009) and the SNP is located in a gene affected by MPH administration (Amiri et al. 2013).
SLC6A2/NET	rs28386840	T allele has been associated with a better MPH response in Korean children (Kim et al. 2010)
SLC6A2/NET	rs192303	The SNP was nominally associated with MPH effect in a GWAS conducted in children (Mick et al. 2008) and is located in a key pharmacological target of MPH.
CES1	rs2244613	CES1 is the main metabolizing enzyme of MPH (Sun et al. 2004) and the SNP has been associated with changes in serum concentrations of the anticoagulant dabigatran also metabolized by CES1 (Pare et al. 2013).
SNAP25	rs363020	A candidate SNP that has not been shown to be associated with MPH response in adult ADHD, but was still labeled as a strong candidate in the same article (Contini et al. 2012).
SNAP25	rs3746544	The T allele has been associated with better MPH dose-response in children (McGough et al. 2006). A study in adults has not shown any effect (Contini et al. 2012)
SNAP25	rs1051312	The T allele has been associated with worse MPH response in children (McGough et al. 2006)
COMT	rs4680 (Val158Met)	The G allele (Val) has been associated with a better response to MPH in children (Cheon et al. 2008; Kereszturi et al. 2008), though not in adults (Contini et al. 2012).
SNP = Single nucleotide polymorphism, GWAS = Genome-wide association study, MPH = Methylphenidate, UTR = Untranslated region , VNTR = Variable number tandem repeat		

Supplementary table 2. Summary of the genotyping per genotyping method

Genotyping platform	MPH responders	MPH non-responders	Total
Sequenom MassARRAY iPLEX	423	66	489
Illumina HumanExome BeadChip 12v1_B	432	66	498
VNTR fragment analysis	476	77	553
Unique patients	487	77	564
VNTR = Variable number tandem repeat			

Supplementary table 3. Results from statistical analysis

Gene	SNP	Risk allele	Observed frequency of risk allele	Additive model		Dominant model	
				OR (95% CI)	p-value*	OR (95% CI)	p-value*
GRM7	rs3792452	A	0.144	0.814 (0.490 -1.354)	0.428	0.706 (0.403 - 1.236)	0.223
DRD5	18.5 kb 5-prime VNTR	148bp	0.521	0.971 (0.690 -1.365)	0.864	1.190 (0.682 -2.075)	0.540
LPHN3	rs6551665	G	0.397	1.037 (0.711 -1.511)	0.851	1.100 (0.641 - 1.888)	0.730
LPHN3	rs6858066	A	0.491	1.188 (0.820 -1.722)	0.363	1.187 (0.664 -2.124)	0.563
LPHN3	rs2345039	G	0.423	0.945 (0.650 -1.375)	0.768	0.813 (0.457 -1.444)	0.479
SLC6A3/DAT1	rs2963238	A	0.456	0.984 (0.671 - 1.441)	0.933	1.092 (0.617 -1.933)	0.763
SLC6A3/DAT1	rs2652511	T	0.454	1.039 (0.711 -1.518)	0.845	1.220 (0.698 -2.132)	0.485
SLC6A3/DAT1	3'UTR VNTR	10 repeat	0.706	1.077 (0.747 -1.554)	0.690	0.772 (0.315 -1.893)	0.572
ADRA2A	**rs1800544 (MspI)**	**G**	**0.274**	0.728 (0.482 -1.100)	0.131	**0.560 (0.329 -0.953)**	**0.033**
ADRA2A	rs553668 (DRAI)	T	0.181	0.719 (0.441 -1.173)	0.186	0.628 (0.368 -1.072)	0.088
DRD4	Exon 3 VNTR	7 repeat	0.193	0.983 (0.630 -1.534)	0.939	1.093 (0.652 -1.834)	0.736
BDNF	rs6265	A	0.194	1.159 (0.718 -1.872)	0.546	1.071 (0.615 -1.865)	0.808
BDNF	rs61888800	T	0.289	0.785 (0.530 -1.160)	0.224	0.876 (0.518 -1.482)	0.621
SLC6A2/NET	rs28386840	T	0.240	1.377 (0.861 -2.203)	0.181	1.402 (0.812 -2.420)	0.226
SLC6A2/NET	rs192303	C	0.309	0.988 (0.666 -1.466)	0.954	0.791 (0.466 -1.342)	0.384
CES1	rs2244613	C	0.188	0.970 (0.611 -1.539)	0.897	1.057 (0.603 - 1.855)	0.846
SNAP25	rs363020	T	0.129	1.565 (0.846 -2.897)	0.154	1.669 (0.837 -3.325)	0.146
SNAP25	rs3746544	C	0.338	0.934 (0.638 -1.368)	0.726	0.913 (0.536 -1.555)	0.738

SNAP25	rs1051312	C	0.306	0.946 (0.634 -1.412)	0.785	0.915 (0.542 -1.545)	0.740
COMT	rs4680	G	0.465	1.315 (0.906 -1.908)	0.150	1.072 (0.606 -1.896)	0.812

*p-values presented are uncorrected. The Bonferroni corrected significance threshold is p = 0.00125.

SNP = Single Nucleotide Polymorphism, OR = Odds ratio, VNTR = Variable Number Tandem Repeat, 95% CI = 95% confidence interval.

OR > 1 means that the variant is associated with MPH response and OR < 1 that the variant is associated with MPH non-response.

References

Amiri A, Torabi Parizi G, Kousha M, Saadat F, Modabbernia MJ, Najafi K, Atrkar Roushan Z (2013) Changes in plasma Brain-derived neurotrophic factor (BDNF) levels induced by methylphenidate in children with Attention deficit-hyperactivity disorder (ADHD) Progress in neuro-psychopharmacology & biological psychiatry 47:20-24 doi:10.1016/j.pnpbp.2013.07.018

Arcos-Burgos M et al. (2010) A common variant of the latrophilin 3 gene, LPHN3, confers susceptibility to ADHD and predicts effectiveness of stimulant medication Molecular psychiatry 15:1053-1066 doi:10.1038/mp.2010.6

Cheon KA, Cho DY, Koo MS, Song DH, Namkoong K (2009) Association between homozygosity of a G allele of the alpha-2a-adrenergic receptor gene and methylphenidate response in Korean children and adolescents with attention-deficit/hyperactivity disorder Biological psychiatry 65:564-570 doi:10.1016/j.biopsych.2008.12.003

Cheon KA, Jun JY, Cho DY (2008) Association of the catechol-O-methyltransferase polymorphism with methylphenidate response in a classroom setting in children with attention-deficit hyperactivity disorder International clinical psychopharmacology 23:291-298 doi:10.1097/YIC.0b013e328306a977

Cheon KA, Kim BN, Cho SC (2007) Association of 4-repeat allele of the dopamine D4 receptor gene exon III polymorphism and response to methylphenidate treatment in Korean ADHD children Neuropsychopharmacology : official publication of the American College of Neuropsychopharmacology 32:1377-1383 doi:10.1038/sj.npp.1301244

Contini V et al. (2012) No significant association between genetic variants in 7 candidate genes and response to methylphenidate treatment in adult patients with ADHD Journal of clinical psychopharmacology 32:820-823 doi:10.1097/JCP.0b013e318270e727

Contini V et al. (2011) Adrenergic alpha2A receptor gene is not associated with methylphenidate response in adults with ADHD European archives of psychiatry and clinical neuroscience 261:205-211 doi:10.1007/s00406-010-0172-4

da Silva TL, Pianca TG, Roman T, Hutz MH, Faraone SV, Schmitz M, Rohde LA (2008) Adrenergic alpha2A receptor gene and response to methylphenidate in attention-deficit/hyperactivity disorder-predominantly inattentive type Journal of neural transmission (Vienna, Austria : 1996) 115:341-345 doi:10.1007/s00702-007-0835-0

de Azeredo LA et al. (2014) Further evidence for the association between a polymorphism in the promoter region of SLC6A3/DAT1 and ADHD: findings from a sample of adults European archives of psychiatry and clinical neuroscience doi:10.1007/s00406-014-0486-8

Froehlich TE et al. (2011) Pharmacogenetic predictors of methylphenidate dose-response in attention-deficit/hyperactivity disorder Journal of the American Academy of Child and Adolescent Psychiatry 50:1129-1139 e1122 doi:10.1016/j.jaac.2011.08.002

Hamarman S, Fossella J, Ulger C, Brimacombe M, Dermody J (2004) Dopamine receptor 4 (DRD4) 7-repeat allele predicts methylphenidate dose response in children with attention deficit hyperactivity disorder: a pharmacogenetic study Journal of child and adolescent psychopharmacology 14:564-574 doi:10.1089/cap.2004.14.564

Hong SB, Kim JW, Cho SC, Shin MS, Kim BN, Yoo HJ (2012) Dopaminergic and noradrenergic gene polymorphisms and response to methylphenidate in korean children with attention-deficit/hyperactivity disorder: is there an interaction? Journal of child and adolescent psychopharmacology 22:343-352 doi:10.1089/cap.2011.0076

Kambeitz J, Romanos M, Ettinger U (2014) Meta-analysis of the association between dopamine transporter genotype and response to methylphenidate treatment in ADHD The pharmacogenomics journal 14:77-84 doi:10.1038/tpj.2013.9

Keresztur E et al. (2008) Catechol-O-methyltransferase Val158Met polymorphism is associated with methylphenidate response in ADHD children American journal of medical genetics Part B, Neuropsychiatric genetics : the official publication of the International Society of Psychiatric Genetics 147B:1431-1435 doi:10.1002/ajmg.b.30704

Kim BN et al. (2011) Val/Val genotype of brain-derived neurotrophic factor (BDNF) Val(6)(6)Met polymorphism is associated with a better response to OROS-MPH in Korean ADHD children The international journal of neuropsychopharmacology / official scientific journal of the Collegium Internationale Neuropsychopharmacologicum (CINP) 14:1399-1410 doi:10.1017/s146114571100099x

Kim BN, Kim JW, Hong SB, Cho SC, Shin MS, Yoo HJ (2010) Possible association of norepinephrine transporter -3081(A/T) polymorphism with methylphenidate response in attention deficit hyperactivity disorder Behavioral and brain functions : BBF 6:57 doi:10.1186/1744-9081-6-57

Labbe A, Liu A, Atherton J, Gizenko N, Fortier ME, Sengupta SM, Ridha J (2012) Refining psychiatric phenotypes for response to treatment: contribution of LPHN3 in ADHD American journal of medical genetics Part B, Neuropsychiatric genetics : the official publication of the International Society of Psychiatric Genetics 159B:776-785 doi:10.1002/ajmg.b.32083

Licinio J, Dong C, Wong ML (2009) Novel sequence variations in the brain-derived neurotrophic factor gene and association with major depression and antidepressant treatment response Archives of general psychiatry 66:488-497 doi:10.1001/archgenpsychiatry.2009.38

McGough J et al. (2006) Pharmacogenetics of methylphenidate response in preschoolers with ADHD Journal of the American Academy of Child and Adolescent Psychiatry 45:1314-1322 doi:10.1097/01.chi.0000235083.40285.08

Mick E, Neale B, Middleton FA, McGough JJ, Faraone SV (2008) Genome-wide association study of response to methylphenidate in 187 children with attention-deficit/hyperactivity disorder American journal of medical genetics Part B, Neuropsychiatric genetics : the official publication of the International Society of Psychiatric Genetics 147B:1412-1418 doi:10.1002/ajmg.b.30865

Pare G et al. (2013) Genetic determinants of dabigatran plasma levels and their relation to bleeding Circulation 127:1404-1412 doi:10.1161/circulationaha.112.001233

Park S, Kim JW, Kim BN, Hong SB, Shin MS, Yoo HJ, Cho SC (2013) No significant association between the alpha-2A-adrenergic receptor gene and treatment response in combined or inattentive subtypes of attention-deficit hyperactivity disorder Pharmacopsychiatry 46:169-174 doi:10.1055/s-0033-1343485

Sun Z et al. (2004) Methylphenidate is stereoselectively hydrolyzed by human carboxylesterase CES1A1 The Journal of pharmacology and experimental therapeutics 310:469-476 doi:10.1124/jpet.104.067116
Tahir E, Yazgan Y, Cirakoglu B, Ozbay F, Waldman I, Asherson PJ (2000) Association and linkage of DRD4 and DRD5 with attention deficit hyperactivity disorder (ADHD) in a sample of Turkish children Molecular psychiatry 5:396-404
Xu M et al. (2010) Pharacogenetic effects of dopamine transporter gene polymorphisms on response to chlorpromazine and clozapine and on extrapyramidal syndrome in schizophrenia Progress in neuro-psychopharmacology & biological psychiatry 34:1026-1032 doi:10.1016/j.pnpbp.2010.05.017
